# Strong Antimicrobial Effects of Xanthohumol and Beta-Acids from Hops against *Clostridioides difficile* Infection In Vivo

**DOI:** 10.3390/antibiotics10040392

**Published:** 2021-04-06

**Authors:** Radek Sleha, Vera Radochova, Alexander Mikyska, Milan Houska, Radka Bolehovska, Sylva Janovska, Jaroslav Pejchal, Lubica Muckova, Pavel Cermak, Pavel Bostik

**Affiliations:** 1 Department of Epidemiology, Faculty of Military Health Sciences, University of Defence, 500 03 Hradec Kralove, Czech Republic; radek.sleha@unob.cz (R.S.); vera.radochova@unob.cz (V.R.); sylva.janovska@unob.cz (S.J.); 2 Research Institute of Brewing and Malting, 110 00 Prague, Czech Republic; mikyska@beerresearch.cz; 3 Food Research Institute, 110 00 Prague, Czech Republic; milan.houska@vupp.cz; 4 Institute of Clinical Microbiology, University Hospital, 500 03 Hradec Kralove, Czech Republic; radka.bolehovska@fnhk.cz; 5 Department of Toxicology and Military Pharmacy, Faculty of Military Health Sciences, University of Defence, 500 03 Hradec Kralove, Czech Republic; jaroslav.pejchal@unob.cz (J.P.); Lubica.Muckova@unob.cz (L.M.); 6 Thomayer Hospital, 110 00 Prague, Czech Republic; pavel.cermak@ftn.cz; 7 Department of Clinical Microbiology, Faculty of Medicine in Hradec Kralove, Charles University, 500 03 Hradec Kralove, Czech Republic

**Keywords:** hops, *C. difficile*, infection, rat model

## Abstract

*Clostridioides* (*C.*) *difficile* is an important causative pathogen of nosocomial gastrointestinal infections in humans with an increasing incidence, morbidity, and mortality. The available treatment options against this pathogen are limited. The standard antibiotics are expensive, can promote emerging resistance, and the recurrence rate of the infection is high. Therefore, there is an urgent need for new approaches to meet these challenges. One of the possible treatment alternatives is to use compounds available in commonly used plants. In this study, purified extracts isolated from hops—alpha and beta acids and xanthohumol—were tested in vivo for their inhibitory effect against *C. difficile*. A rat model of the peroral intestinal infection by *C. difficile* has been developed. The results show that both xanthohumol and beta acids from hops exert a notable antimicrobial effect in the *C. difficile* infection. The xanthohumol application showed the most pronounced antimicrobial effect together with an improvement of local inflammatory signs in the large intestine. Thus, the hops compounds represent promising antimicrobial agents for the treatment of intestinal infections caused by *C. difficile*.

## 1. Introduction

*C. difficile* (formerly *Clostridium difficile*) is an anaerobic, spore-forming Gram-positive bacterium, which is widely found in the mammalian gastrointestinal tract (GIT), including in humans. Its growth is under physiological circumstances suppressed by the intestinal microbiome. One of the main virulence factors of *C. difficile* is the ability to form aerotolerant spores allowing bacteria to persist within the host and to disseminate by patient-to-patient contact or environmental contamination. Clostridial toxins A and B represent other important pathogenetic factors. These exotoxins have enterotoxic and cytotoxic activity that cause primary symptoms of the disease [1,2,3].

*C. difficile* is opportunistic bacteria, a pathological role which usually manifests in hospital settings in patients with antibiotic treatment that alter the colonic microbiome. The extensive antibiotic resistance of *C. difficile* leads to its proliferation in the colon and toxin production. The other patient-related risk factors affecting this process are advanced age, increased severity of underlying illness, prior hospitalization, use of feeding tubes, gastrointestinal surgery, and therapy using proton-pump inhibitors. Clinical symptoms of *C. difficile* infection manifest by signs ranging from mild diarrhea to pseudomembranous colitis, toxic megacolon, bowel perforation, and sepsis [4,5].

The treatment strategy of the *C. difficile*-induced infection depends on the health status of the patient and comorbidities. The first step in treating *C. difficile* is to discontinue the antibiotic therapy that triggered the *C. difficile* overgrowth leading to symptoms or, if necessary, to replace the antibiotic with another one. In intermediate and severe cases, antibiotics remain the recommended treatment. Vancomycin and metronidazole are the antibiotics of choice. They are the most efficient ones in clinical practice. The other drugs utilized in such cases include fidaxomicin, tigecycline, or teicoplanin. Recurrent *C. difficile* infections occur in up to 35% of cases due to the relapse of infection or reinfection with another strain [6,7,8,9]. Preserving physiological microbiome and microbial diversity in the gastrointestinal tract may prevent or even treat the disease. Other potential therapeutical modalities represent non-antibiotic therapies, such as the application of probiotics, intravenous immunoglobulins, and fecal transplants [2,5,10].

However, the commonly used antibiotic therapy could induce bacterial resistance and create a burden for the patient. Therefore, effective non-antibiotic alternatives are urgently needed and have been a focus of research for several years, including plant derivatives. Among those, various hops extracts and individual hops compounds have been known for some time for their antimicrobial activity. Strong antimicrobial activity of hops compounds (isolated from *Humulus lupulus* L.), including xanthohumol or alfa- and beta-bitter acids, has been reported against *C. difficile* in vitro. With minimal inhibitory concentrations being close to commonly used antibiotics, these compounds may represent a potential alternative for treating *C. difficile* infections [11,12,13,14,15,16].

Various animals pretreated with antibiotic regimens followed by oral challenge with *C. difficile* have been used as suitable models for *C. difficile*-induced disease [1,5,17,18,19,20].

The aim of this study was to determine the antibacterial properties of pure hops extracts of alpha- and beta-bitter acids and xanthohumol in *C. difficile* infection in vivo. For this purpose, a rat model of infection was developed.

## 2. Results

To evaluate the in vivo antibacterial effect of purified hops compounds, the experimental animals were first conditioned using an antibiotic regimen to clear their intestines from microflora and the endogenous *C. difficile* infection. The animals were then experimentally infected with a quantified dose of ribotyped hypervirulent *C. difficile* strain. Several different bacterial isolates were first tested for their pathogenicity in rats leading to the selection of the most pathogenic one (data not shown). A total of 35 animals divided into five experimental groups were then monitored for both the general signs of *C. difficile*-induced disease and for the presence of the bacteria in feces. Prior to the infection of animals with the experimental strain, only two animals tested positive for the endogenous colonization with *C. difficile*. Both of them belonged to the group subsequently treated with beta-bitter acids. One of these rats died during the experiment. Within three days post-infection, all animals were culture positive for the presence of vegetative form or spores of *C. difficile*. The general clinical symptoms of *C. difficile* infection were also observed in all animals (apathy, bristle coat). At this time point, the administration of antibacterial substances started (xanthohumol, beta-acids, xanthohumol + beta-acids, or vancomycin), except for the control group which received no treatment. Alpha bitter acids were not included in this study as they showed only a limited effectivity in vitro [13] and no effect in a preliminary experiment in vivo (data not shown).

**Figure 1:**
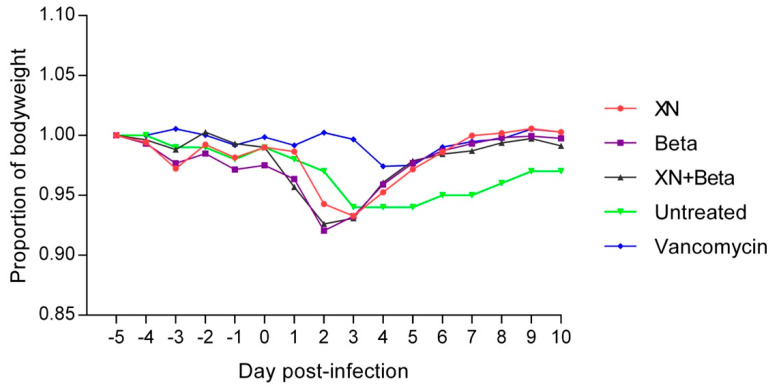
The proportion of body weight of infected rats during the *C. difficile* infection. Data are presented as means of the animal body weights at the individual time points relative to their body weight at the onset of the experiment (day-5). The animals were infected on day 0, and treatments started on day 3. XN—xanthohumol, Beta—beta-acids.

The body weight of each animal was monitored during the entire experiment. Until day 3 post-infection, all infected animals suffered weight loss, and several rats also exhibited symptoms of diarrhea. After the onset of antimicrobial treatment, the body weight of each treated animal started to improve rapidly. As illustrated in Figure 1, all tested hops compounds and vancomycin effectively stopped further weight loss of animals and led to the normalization of their body weight compared to the untreated control group. All treated groups showed significant differences in body weight on days 7, 8, and 9 (*p* < 0.05) compared to the untreated control. The most profound and rapid positive effect of the hops compound was observed especially in the beta-bitter acid group. This was despite the fact that the animals in all three hops compound-treated groups exhibited the most pronounced weight loss after the infection but before the treatment. The other clinical symptoms in animals in the treated groups improved as well.

**Figure 2:**
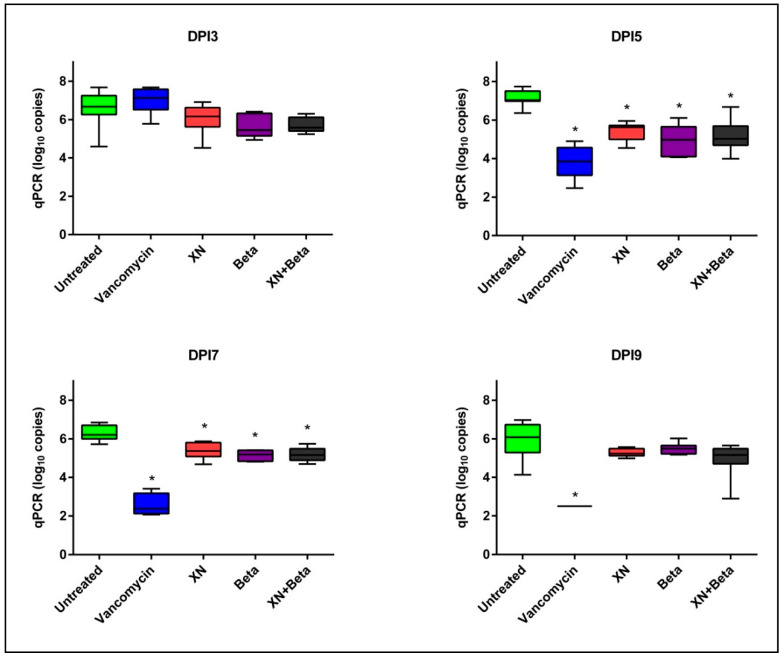
Bacterial load (log_10_ copies per reaction) of *C. difficile* per g of stool from rats. * Significant differences compared to untreated controls (*p* < 0.05). DPI—day post-infection, XN—xanthohumol, Beta—beta-acids.

The quantitative determination of *C. difficile* in fecal samples showed the ability of tested compounds to reduce the bacterial load (Figure 2). During the treatment period (samples collected on days 3, 5, 7, and 9 post-infection), all three hops-compound treatment modalities showed similar inhibitory effects when compared to the untreated control. The reduction of the bacterial load was observed from day 3 post-infection. In all three treatment groups the differences in bacterial load of *C. difficile* were significant (*p* < 0.05) when compared to the untreated controls. Treatment by vancomycin in the positive control group showed a rapid decline of the bacterial load from day 5 (Figure 2).

**Figure 3:**
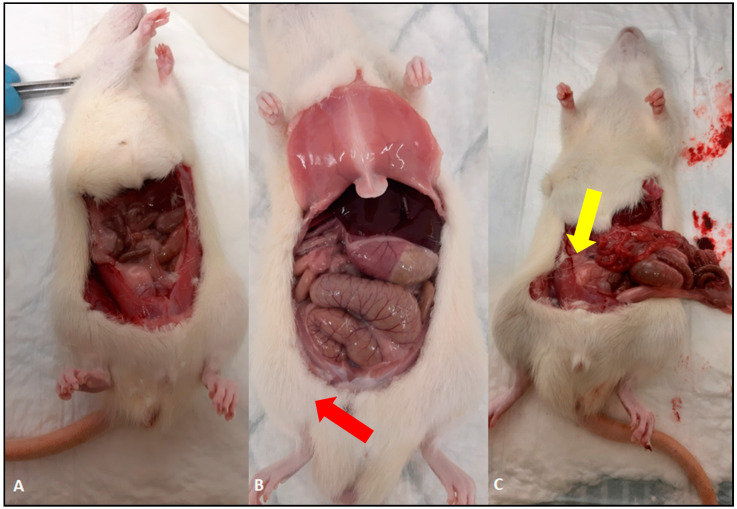
Macroscopical evaluation of *C. difficile* infection in the large and small intestines. (**A**) Normal macroscopical finding in the Xanthohumol treated rat; (**B**) edema in the large intestine (red arrow) and (**C**) hyperemia in the small intestine in infected untreated rats (yellow arrow).

We further evaluated the effect of treatments on the infection in intestines in experimental animals both at the macroscopical and microscopical levels after the termination of the experiment. The macroscopical evaluation showed marked hyperemia and swelling of the bowel in the animals from the untreated group. All the administered treatments led to a physiological bowel appearance after the termination of the experiment. Representative examples are shown in Figure 3.

**Figure 4:**
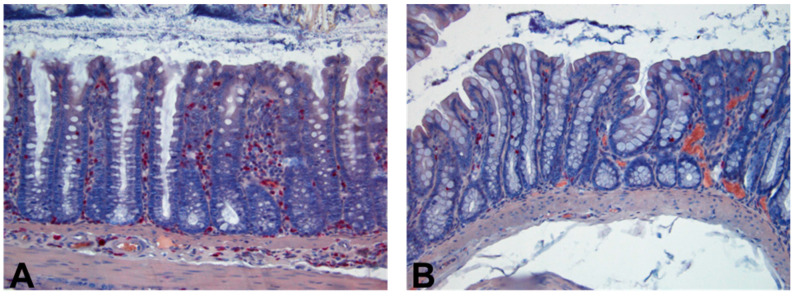
Histopathological changes in the colon from rats infected with *C. difficile* 10 days post-infection (**A**) with no treatment and (**B**) treated with xanthohumol. Tissue samples are stained with a naphthol AS-D chloroacetate kit and counterstained with hematoxylin (200-fold original magnification). *C. difficile* infection leads to high neutrophil infiltration (red color) and edema (**A**), which is substantially reduced by the xanthohumol treatment (**B**).

The histopathological samples were collected at the end of the experiment (day 10). Microscopical examination showed *C. difficile* infection-induced edema and leukocyte infiltration in the large intestine of untreated animals. These findings were significantly reduced by all treatments (examples shown in Figure 4).

**Figure 5:**
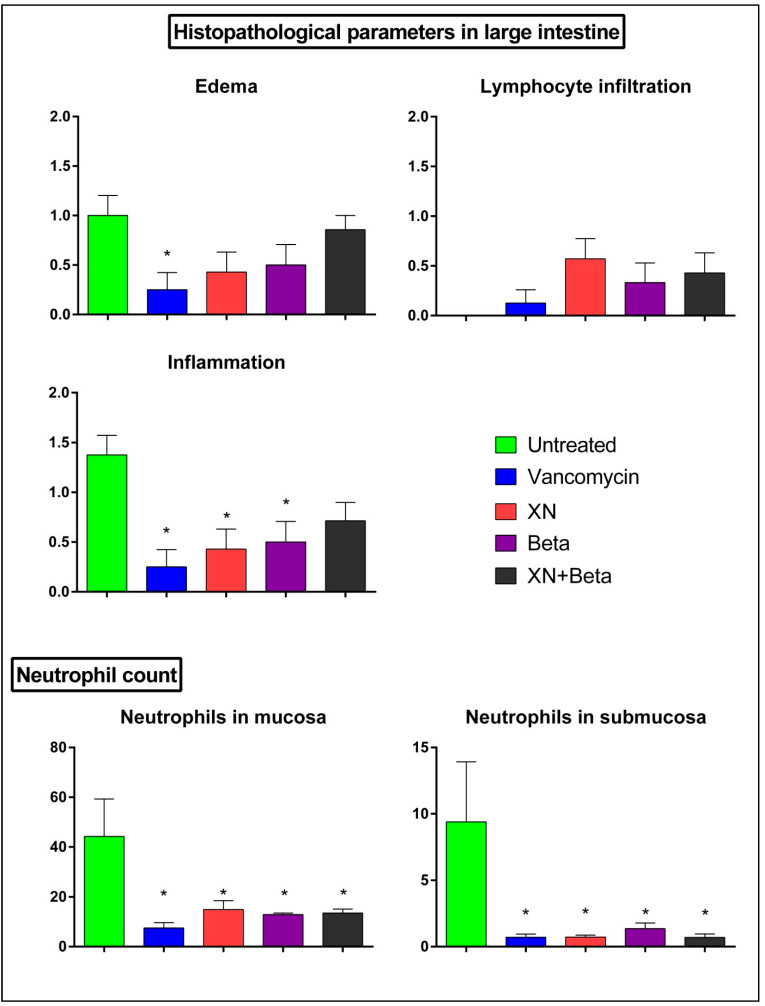
Histopathological analysis of the effect of treatments in large intestines of rats in the individual cohorts. Data are presented as mean ± standard deviation. * indicates significant differences compared to untreated controls (*p* < 0.05).

Further analysis showed (Figure 5) that treatments with xanthohumol or beta-bitter acids reduced the edema in the large intestine. Additionally, the individual compounds significantly reduced the histopathological score of inflammation. Quantitation of neutrophils in the mucosal and submucosal tissues showed that all treatment modalities led to significant decreases in the numbers of neutrophils per microscopic field.

The extent of histopathological changes in the small intestine of infected rats was generally low, even in untreated controls (data not shown).

Taken together, xanthohumol and beta-bitter acids show a clear antimicrobial effect against *C. difficile* infection in vivo, leading to both notable decreases in bacterial load (significant especially at days 5 and 7 post-infection) and normalization of inflammatory markers in the mucosal and submucosal tissues of the large intestine. The best antimicrobial effects in this model in vivo are obtained with the administration of either xanthohumol alone or a mixture of xanthohumol and beta-bitter acids from hops. In addition, the developed and presented animal model in rats provides a useful tool in studies of the pathogenesis and therapy of colitis induced by *C. difficile* infection.

## 3. Discussion

*C. difficile* is one of the most common nosocomial pathogens with a worldwide distribution. It has been associated with pseudomembranous colitis, often leading to life-threatening diarrhea with increased morbidity and mortality rates. The currently used therapeutic management of *C. difficile* infection is achieved by the termination of “unnecessary” antibiotics that may lead to the development of the condition and administration of antimicrobials effective against *C. difficile*. The currently used ones are represented by vancomycin and metronidazole. However, the infection has a relatively high recurrence rate. The recurrent disease and fulminant courses require an intra-intestinal or parenteral administration of antibiotics. Some drugs, such as metronidazole, are characterized by a high resorption rate and present with serious side effects. Thus, there is an ongoing search for alternative therapies for this disease. One of those is, for example, the fecal microbial transplantation [9,21,22].

Another possible approach explored is the use of compounds with antimicrobial effects available in commonly used plants. Numerous studies have shown that the hops cones represent an abundant source of components with apparent antimicrobial effects against certain bacteria, viruses, fungi, and protozoa. Some of the specific properties for using these hops derivatives as therapeutics is their low cytotoxicity, specifically for the use in the GIT, and their very low adsorption. These features lead to their safety and the absence of side effects. The antibacterial properties of some purified compounds from hops, namely xanthohumol and beta-bitter acids, were determined in vitro for many pathogens, including *C. difficile*, in the past [13].

In the present study, the efficiency of the treatment with two compounds isolated from hops and their mixture was tested in an animal model of the *C. difficile*-induced gastrointestinal infection. We first successfully established the rat model for the *C. difficile* bowel infection. In this model, the bacteria from the hypervirulent human strain were atraumatically introduced into the GIT of animals pre-conditioned with a regimen of antibiotics. In contrast to the hamster model historically used for *C. difficile* infection, the course of the infection in rats is not fulminant and lethal, which allows for an extended antimicrobial efficacy testing. Thus, the rat model is more similar to the disease course of *C. difficile* bowel infection in humans, which will allow not only for in vivo testing of novel antimicrobials but potentially for studies of disease pathogenesis as well.

The results of this study show that all the hops derivatives tested possess antimicrobial properties against the *C. difficile* infection in vivo. However, the pure xanthohumol exhibited higher antimicrobial potential in our model than the other hops compounds tested. It significantly decreased the bacterial load of *C. difficile* in fecal samples after two days of application. These results further corroborate our previously published data of the high in vitro activity of xanthohumol against *C. difficile* determined by the broth dilution method. Similar antimicrobial effects were observed for the combination of xanthohumol and beta-bitter acids. Treatments by these substances also had positive effects on body weight and other general signs of the disease in experimental animals compared to the untreated control.

Finally, this work illustrates the antimicrobial potential of the tested compounds against *C. difficile* in in vivo testing. These results show that namely xanthohumol has the potential of being developed into an antimicrobial treatment regimen or to be used in combination with standardly used drugs. The advantage of xanthohumol for such use is its minimal resorption in the intestine, thus allowing for the administration of large doses with no or minimal side effects.

Mechanisms underlying antimicrobial activity of hops-derived compounds have not been extensively studied. Several reports suggest that these compounds affect bacterial cell membrane integrity, interfere with fatty acid metabolism, and lead to an accumulation of protons intracellularly and subsequent cell starvation [23,24,25]. A combination of these mechanisms may underlie effects of these compounds against *C. difficile* observed in the presented study. However, the elucidation of the exact mechanism will need further investigation. Taken together, the results show the purified substances from hops are promising candidates for further development and use in difficult-to-treat infections in humans, such as colitis caused by *C. difficile*.

## 4. Materials and Methods

### 4.1. Hops Compounds

A pure isolate of beta-acids was prepared at the Hop Research Institute in Zatec and further purified at the Research Institute of Brewing and Malting according to the procedure described by Krofta et al. [26]. The first step involved partitioning the CO_2_ hops extract solution in an alkaline medium of sodium carbonate and sodium hydroxide to separate the alpha-acids and beta-acids fractions. In the next step, the crude beta-fraction has been used for the isolation of pure beta-acids (99.7% *w*/*w*) through crystallization from the solvent mixture. The isolate of xanthohumol (84.3% *w*/*w*) was prepared following the procedure described by Biendl [27]. The process consists of the selective sorption of prenylflavonoids from ethanolic hops extract on polyvinylpyrrolidone. The isolate contains, in addition to xanthohumol, the whole spectrum of different hops prenylflavonoids. A stock solution with a concentration of xanthohumol or beta-acids of 100 mg/1 mL was prepared by dissolving the isolates in dimethyl sulfoxide.

### 4.2. Bacterial Strain and Culture Conditions

The bacterial strain of *C. difficile* used in this study was from the collection of isolates of the Department of Medical Microbiology of Thomayer Hospital in Prague (Czech Republic). The hypervirulent strain 176 was isolated and ribotyped at the Institute of Microbiology, University Hospital and Second Medical Faculty, Charles University in Prague. The ribotype analysis was performed using PCR ribotyping and detection of the presence of toxin production governing genes (tcdA (A), tcdB (B), cdtA, and cdtB (binary)) was performed by a multiplex PCR. The strain was cultured on selective *C. difficile* blood agar (LabMediaServis, Jaromer, Czech Republic) supplemented with norfloxacin (12 µg/mL) and moxalactam (32 µg/mL). Bacterial stocks for cryopreservation were prepared on porous beads (ITEST, Hradec Kralove, Czech Republic). For each experiment, fresh bacterial culture was prepared as follows. The porous bead with *C. difficile* was inoculated onto the agar plate. The culture was performed under anaerobic conditions using an anaerobic gas chamber and an AnaeroGen sachet (Oxoid, Basingstoke, UK) at 37 °C for 48 h.

### 4.3. Animals and Housing

Male Wistar rats (weight 330–460 g) were purchased from VELAZ (Prague, Czech Republic). The animals were housed under veterinary control and standard conditions (light cycle 12 h/12 h, standard laboratory diet, and water ad libitum). All the experiments were performed with permission and under the supervision of the Ethics Committee of the Faculty of Military Health Sciences (Hradec Kralove, Czech Republic).

### 4.4. Experimental Model

**Figure 6:**
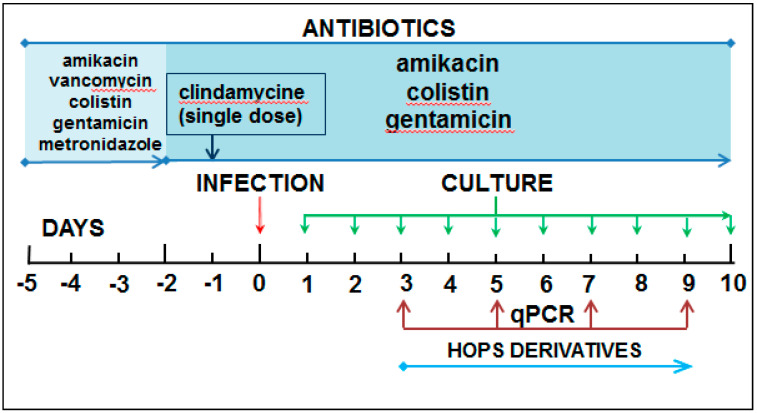
Time course of the experiment.

The experimental animals were subjected to the following protocol to establish a suitable animal model for testing the compound’s in vivo antibacterial activity. On day 5, water containing antibiotic mixture, consisting of amikacin (9.66 mg/kg, Braun Medical, Prague, Czech Republic), colistin (4.2 mg/kg, Teva Pharmaceuticals, Prague, Czech Republic), gentamicin (3.5 mg/kg, LONZA, Basel, Switzerland), metronidazole (21.5 mg/kg, Braun Medical, Prague, Czech Republic), and vancomycin (4.5 mg/kg, Mylan SAS, Saint Priest, France), was given to each animal by intragastric gavage in a total volume of 1 mL. This was followed with identical doses of amikacin, gentamicin, and colistin at day 2. A single dose of clindamycin (10 mg/kg; Fresenius Cabi, Germany) was given intraperitoneally at day 1. On day 0, the stool samples were sampled before the experimental bacterial infection to screen for any endogenous colonization with *C. difficile*. Then each rat was administered with a single dose of *C. difficile* suspension (3 × 10^8^ cells in 1 mL) by intragastric gavage. Rats were then monitored every day for general signs of infection (diarrhea, weight loss, and infection symptoms), and stool samples were collected for *C. difficile* identification until day 10 post-infection by culture and quantification at day 3, 5, 7, 9 post-infection by qPCR (Figure 6). For the evaluation of antibacterial effects of hops compounds, the animals were divided into 5 experimental groups of 7 animals each. There were two control groups: animals in the negative control group I received no antibacterial treatment, while those in the positive control group II received vancomycin (150 mg/L, Mylan SAS, St. Priest, France) as the “standard” antibiotic used in the treatment of pseudomembranous colitis. Animals in groups III, IV, and V were treated with xanthohumol, beta-bitter acids, or a mixture of both substances at the concentration 5 mg/kg, respectively. All antibacterial agents were administered every day by intragastric gavage from day 3 post-infection. On day 10 post-infection, the animals were euthanized, and samples from the small and large intestine were collected for further histological examination.

### 4.5. DNA Isolation and Real-Time PCR

The DNA from stool samples of rats was isolated by HigherPurity Stool DNA Isolation kit (Canvax Biotech S.L., Córdoba, Spain) according to the manufacturer’s instructions. DNA samples were then stored at −20 °C until qPCR analysis.

The qPCR assay was performed using *C. difficile* Genesig Advanced Kit (PrimerDesign, Camberley, UK). The amplification reactions were carried out in a final volume of 20 μL, which consisted of 5 μL of the template and 15 μL of the master mix, containing Oasig 2X qPCR Master Mix, 300 nM of each primer and 150 nM fluorescence-labeled probe and distilled water. The assay was performed with the CFX Touch Real-Time PCR Detection System (Biorad, Hercules, CA, USA). The amplification conditions consisted of an initial enzyme activation at 95 °C for 2 min, followed by 50 cycles of denaturation at 95 °C for 10 s, primer annealing at 54 °C for 30 s, and data collection at 60 °C for 1 min. Fluorogenic data was collected through the FAM and VIC channels.

### 4.6. Histopathology Examination

Collected samples were fixed in 10% neutral buffered formalin (Bamed, Ceske Budejovice, Czech Republic). Subsequently, they were histologically processed and stained with hematoxylin and eosin (both from Merck, Kenilworth, NJ, USA), according to Pejchal et al. [28]. The histopathological analysis was performed using a BX-51 microscope (Olympus, Tokyo, Japan) and a semiquantitative scale developer by Shelby et al. [20].

Neutrophil granulocytes were detected using Naphthol AS-D Chloroacetate (specific esterase) Kit (Sigma-Aldrich, St. Louis, MO, USA) according to the manufacturer’s instructions. Naphthol AS-D Chloroacetate positive cells were measured in 6 randomly selected microscopic fields in the mucosal and submucosal compartments at 400fold original magnification on a BX-51 microscope.

### 4.7. Data Analysis

Data obtained by the qPCR assay and weight monitoring were analyzed using Excel (Microsoft^®^ 2010) software or imported into GraphPad Prism 6 (version 6.05, GraphPad Software Inc., San Diego, CA USA) for further analysis. The normality was tested using the Shapiro–Wilk test. Normally distributed data were analyzed using one-way ANOVA with post hoc Student’s *t*-test. Non-normally distributed data were analyzed by Kruskal–Wallis test with post hoc Mann–Whitney test. The differences were considered significant when *p* ≤ 0.05.

## Data Availability

Data available on request due to ethical restrictions.
